# Utility of an artificial intelligence system for classification of esophageal lesions when simulating its clinical use

**DOI:** 10.1038/s41598-022-10739-2

**Published:** 2022-04-23

**Authors:** Ayaka Tajiri, Ryu Ishihara, Yusuke Kato, Takahiro Inoue, Katsunori Matsueda, Muneaki Miyake, Kotaro Waki, Yusaku Shimamoto, Hiromu Fukuda, Noriko Matsuura, Satoshi Egawa, Shinjiro Yamaguchi, Hideharu Ogiyama, Kiyoshi Ogiso, Tsutomu Nishida, Kenji Aoi, Tomohiro Tada

**Affiliations:** 1 Department of Gastrointestinal Oncology, Osaka International Cancer Institute, 3-1-69 Otemae, Chuo-ku, Osaka, 541-8567 Japan; 2 AI Medical Service Inc, Tokyo, Japan; 3 Department of Gastroenterology, Keio University Hospital, Tokyo, Japan; 4 Department of Gastroenterology, Osaka Police Hospital, Osaka, Japan; 5 Department of Gastroenterology, Kansai Rosai Hospital, Hyogo, Japan; 6 Departments of Gastroenterology and Hepatology, Itami City Hospital, Osaka, Japan; 7 Department of Gastroenterology, JR Osaka Railway Hospital, Osaka, Japan; 8 Department of Gastroenterology, Toyonaka Municipal Hospital, Osaka, Japan; 9 Department of Gastroenterology, Kaizuka City Hospital, Osaka, Japan; 10 Tada Tomohiro Institute of Gastroenterology and Proctology, Saitama, Japan; 11 Department of Surgical Oncology, Graduate School of Medicine, The University of Tokyo, Tokyo, Japan

**Keywords:** Oncology, Gastroenterology, Oesophageal cancer

## Abstract

Previous reports have shown favorable performance of artificial intelligence (AI) systems for diagnosing esophageal squamous cell carcinoma (ESCC) compared with endoscopists. However, these findings don’t reflect performance in clinical situations, as endoscopists classify lesions based on both magnified and non-magnified videos, while AI systems often use only a few magnified narrow band imaging (NBI) still images. We evaluated the performance of the AI system in simulated clinical situations. We used 25,048 images from 1433 superficial ESCC and 4746 images from 410 noncancerous esophagi to construct our AI system. For the validation dataset, we took NBI videos of suspected superficial ESCCs. The AI system diagnosis used one magnified still image taken from each video, while 19 endoscopists used whole videos. We used 147 videos and still images including 83 superficial ESCC and 64 non-ESCC lesions. The accuracy, sensitivity and specificity for the classification of ESCC were, respectively, 80.9% [95% CI 73.6–87.0], 85.5% [76.1–92.3], and 75.0% [62.6–85.0] for the AI system and 69.2% [66.4–72.1], 67.5% [61.4–73.6], and 71.5% [61.9–81.0] for the endoscopists. The AI system correctly classified all ESCCs invading the muscularis mucosa or submucosa and 96.8% of lesions ≥ 20 mm, whereas even the experts diagnosed some of them as non-ESCCs. Our AI system showed higher accuracy for classifying ESCC and non-ESCC than endoscopists. It may provide valuable diagnostic support to endoscopists.

## Introduction

Esophageal cancer (EC) is the seventh most common cancer and the sixth most common cause of cancer-related death worldwide^1^. Squamous cell carcinoma (SCC) is the most common subtype, and accounts for 80% of all EC^1^. Advanced esophageal squamous cell carcinoma (ESCC) has a poor prognosis, so detecting and diagnosing it at an early stage is important for a favorable outcome^2–6^. Specifically, it is necessary to correctly differentiate cancerous and non-cancerous abnormal lesions detected by esophagogastroduodenoscopy (EGD).

To detect ESCC, narrow band imaging (NBI) and blue laser imaging (BLI), equipment-based image-enhanced endoscopy, are reportedly useful^7–10^. These are advanced, noninvasive optical techniques that enhance visibility of the superficial structure and microvascular pattern of the esophagus^7^. Previous studies involving NBI and magnification have shown high diagnostic accuracy for esophageal squamous cell carcinoma^7,11–13^, and they are currently regarded as the standard modality for diagnosing esophageal SCC. However, a previous report also showed that the diagnosis of SCCs by NBI and magnification was liable to interobserver variability, and identification accuracy was not very high^14^.

Artificial intelligence (AI) systems have the potential to improve the accuracy of diagnosis by endoscopy. Computer vision deep learning, which is typically based on convolutional neural networks, is the mainstay of recent computer vision AI systems, which have shown good performance in visual tasks. This technology has been applied to the diagnosis of GI cancers, including esophageal SCC^15–17^, and previous studies have shown that AI systems have favorable performance in the detection of ESCC^15,18–20^. In these reports, endoscopists and AI systems used the same magnified still images^15,18^ and video images^19,20^ to diagnose the lesions. In clinical practice, however, endoscopists and AI systems use different methods to make diagnoses. Endoscopists classify lesions comprehensively, based on a variety of images, both magnified and non-magnified images. In contrast, AI systems often use only a few still images for classification, particularly magnified NBI images^15,18^. To evaluate the performance of an AI system as a support tool, it should be evaluated in more realistic situations.

In this study, we compared the performance of our AI system with endoscopists in a situation simulating clinical diagnosis.

## Methods

### Training datasets and image annotation

**Figure 1:**
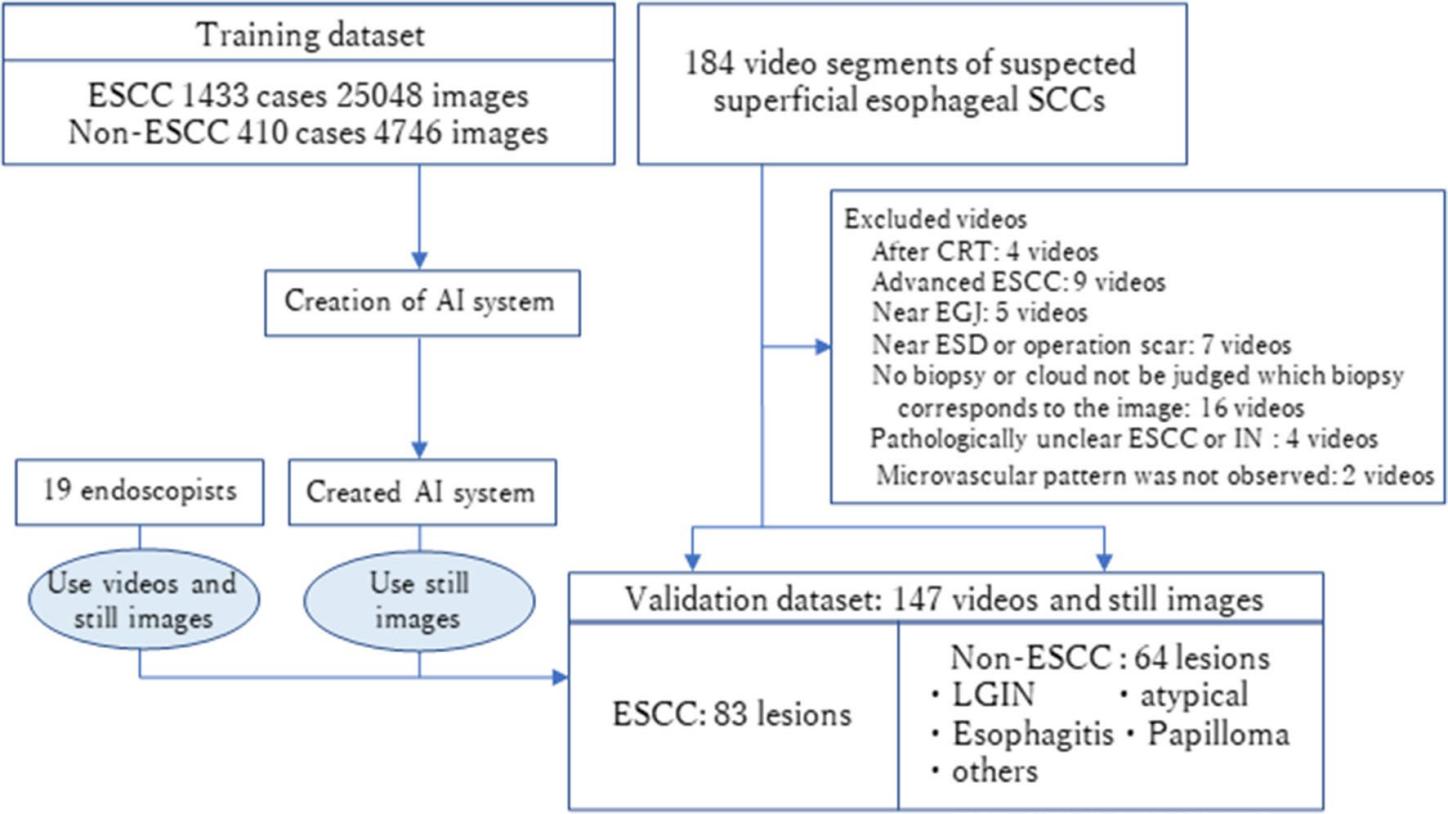
Flowchart of construction of AI and validation dataset. *AI* artificial intelligence, *ESCC* esophageal squamous cell carcinoma, *CRT* chemoradiotherapy, *EGJ* esophagogastric junction, *ESD* endoscopic submucosal dissection, *IN* intraepithelial neoplasia, *LGIN* low-grade intraepithelial neoplasia.

We developed a deep learning-based AI system classification of superficial ESCCs. The system was trained with endoscopic images taken on diagnostic EGD. We gathered endoscopic still and video images of pathologically proven superficial ESCC captured at Osaka International Cancer Institute, Fukuoka University Chikushi Hospital, and Niigata University Hospital between December 2005 and June 2019. We also gathered images of noncancerous lesions and normal esophagi taken at Osaka International Cancer Institute between January 2009 and June 2019. Noncancerous lesions included pathologically or endoscopically diagnosed esophagitis, submucosal tumor, vascular abnormality, glycogenic acanthosis, atypical epithelium, and intraepithelial neoplasia. Poor quality images due to bleeding, halation, or defocus were excluded. As in our previous studies^20,21^, still images extracted from videos were used to diversify cancer images in terms of shooting conditions (e.g. various distances, angles, and focus). The endoscopic procedures were carried out using the following equipment: GIF-RQ260Z, GIF-FQ260Z, GIF-Q240Z, GIF-H290Z, GIF-HQ290, GIF-H260Z, GIF-XP290N, GIF-Q260J, or GIF-H290 endoscopes (Olympus, Tokyo, Japan) with the video processors CV260 (Olympus), EVIS LUCERA CV-260/CLV-260, or EVIS LUCERA ELITE CV-290/CLV-290SL (Olympus Medical Systems); or EG-L590ZW, EG-L600ZW, or EG-L600ZW7 endoscopes (Fujifilm Co, Tokyo, Japan) and the video endoscopic system LASEREO (Fujifilm Co.). For observations that used the LASEREO system, white-light imaging (WLI) and BLI, which provide images similar to NBI, were used. A black soft hood was equipped on the tip of the endoscope to keep an appropriate distance between the tip of the endoscope and esophageal wall during magnified observations. B-mode level 8 for NBI and level 5–6 for BLI was used for the structure enhancement function. After extracting still images from the videos, our training dataset for AI included 25,048 images from 1433 pathologically proven superficial ESCCs and 4746 images from 410 noncancerous lesions and normal esophagi (Fig. 1). These images included those captured by magnified endoscopy (ME) and non-magnified endoscopy (non-ME) with WLI, NBI, and BLI. As in our previous study^20,21^, the images were annotated manually by precisely delineating the boundaries and filling in the areas containing the ESCC or other abnormal lesions. Annotation was conducted by eight endoscopists and all annotated images were reconfirmed by a board-certified trainer (R.I.) at the Japan Gastroenterological Endoscopy Society. While annotating the images, we referred to images captured by various imaging modalities: WLI, NBI/BLI, and chromoendoscopy with and without ME as needed.

#### Construction of the AI system

Big Transfer (BiT), a recipe of pre-training networks in computer vision for effective learning of general features, is expected to advance the performance of computer vision models. According to "Big Transfer (BiT): General Visual Representation Learning"^22^, BiT achieved strong performance on over 20 datasets.

For endoscopic images, the new recipe is expected to work well because the pre-trained model already comes with a good understanding of the visual world. All BiT models consist of a vanilla ResNet-v2 architecture which is then customized. Considering the size of the training dataset and validation accuracy, we adopted a BiT-M (ResNet-101 × 1) for our AI system. This model was pre-trained on the full ImageNet-21k dataset, which is a public dataset containing 14.2 million images and 21k classes organized by the WordNet hierarchy. At the phase of transfer learning, we trained the model using a BiT-HyperRule, which is in the recipe, to select the most important hyperparameters for tuning. We used SGD with an initial learning rate of 0.003 and momentum 0.9. We fine-tuned the model for 3900 steps with a batch size of 32. The learning rate was decayed by a factor of 10 at 30%, 60% and 90% of the training steps. The model was trained on the dataset and validated using the PyTorch deep learning framework^23^, which is one of the most popular and widely used frameworks. For the training dataset, we included endoscopic images with various shooting conditions and resolutions to improve the generalizability of the system. Each image was resized to 512 × 512 pixels for optimal analysis.

### Video images for the validation dataset

To create the independent validation datasets, NBI/BLI endoscopic video segments for the diagnoses of suspected superficial esophageal SCCs were taken by eight endoscopists from December 2019 to July 2020 at the Osaka International Cancer Institute. The video, including the continuous diagnostic procedures, consisted of detecting the lesion by non-ME image, coming close to the lesion, and observing the microvascular pattern by ME image. The videos were directly used as validation videos to avoid any bias derived from editing. All lesions for validation datasets were pathologically confirmed by biopsy specimens or endoscopic submucosal dissection (ESD) specimens. If the diagnoses of the lesion by biopsy and ESD were different, diagnosis by ESD was adopted. Non-cancerous lesions were also histologically confirmed to have no malignancy, including esophagitis, atypical epithelium or papilloma, or low-grade intraepithelial neoplasia. Subjects with the following were excluded: (1) advanced stage of ESCC, (2) a history of chemotherapy or/and radiation therapy for esophagi, (3) lesions near the esophagogastric junction, (4) lesions near ESD or operation scars, (5) inconclusive diagnosis of cancer by pathology.

Regarding sample size, we initially estimated a minimum of 60 ESCCs for the validation set, based on the sensitivity of 90% of the AI system in a previous study^20^. To maximize study power, we sequentially collected as many ESCCs as possible, more than 60 lesions, from consecutive cases during the period to reduce the 95% confidence interval to under 20%. Altogether, 147 lesions from 130 patients, including 83 cancers and 64 noncancers, were collected by nine endoscopists for the validation movie dataset.

### Evaluation of the AI system and endoscopists

The trained neural network generated a diagnosis of esophageal SCC or noncancerous lesions, such as abnormal vessels or esophagitis, with a continuous value between 0 and 1, corresponding to the probability of that diagnosis. We took one still ME image, just like our daily practice, from the validation video dataset. These still images (validation still image dataset) were judged by the AI system as ESCC or non-ESCC.

We invited 19 endoscopists with varying years of experience from six centers to interpret the validation dataset. Thirteen of them were board-certified specialists at the Japan Gastroenterological Endoscopy Society (experts). The median years of experience of endoscopy of all endoscopists was 12 (range 2–25 years) and the median number of EGDs they had performed was 6000 (range 500–25,000), and for the experts only were 15 years (range 9–25) and 10,000 (range 4000–25,000). They had been diagnosing gastrointestinal cancers, including superficial ESCC, in their daily practice. They interpreted the validation video dataset, and the validation still image dataset if needed, and classified them to ESCC or non-ESCC.

### Statistical analysis

The main outcome measures were diagnostic accuracy, sensitivity and specificity for superficial ESCC. These parameters were calculated as follows: Accuracy = correctly diagnosed lesions/total lesions; Sensitivity = the number of correctly detected superficial ESCC/the number of total superficial ESCC; Specificity = the number of correctly diagnosed noncancerous lesions or normal esophagus videos/the number of total noncancers or normal esophagus videos; Positive predictive value = correctly diagnosed ESCC/lesions diagnosed as ESCC. Results are shown as averages (arithmetic mean) and 95% confidence intervals (CI). For the results of AI system, the 95% CI was calculated as the non-asymptotic binomial CI using the number of the total cases and of the correctly diagnosed cases and α = 0.05. Also, for the results of endoscopists, 95% CI of the mean was calculated with normal approximation by producing the unbiased variance and standard error from the means of the rates and calculating the error of the mean. All analyses were performed on a personal computer using the EZR software package, version 1.27 (Saitama Medical Center, Jichi Medical University, Tochigi, Japan).

### Ethics approval

This study was approved by the ethics committee of Osaka International Cancer Institute (no. 18149-5). We confirmed that all experiments were performed in accordance with relevant guidelines and regulations. The movies of endoscopy for constructing the AI system were retrospectively collected, and for validating the AI system was collected in anonymized form and non-interventional way, so informed consent was obtained from all patients by opt-out.

## Results

### Details of validation datasets and endoscopist

A total of 147 videos were taken by eight endoscopists from December 2019 to July 2020. After excluding 31 videos based on the exclusion criteria, 83 ESCC video and 64 non-ESCC videos were included in the validation dataset (Fig. 1). The videos were between 4 and 29 s long, and details of included lesions are shown in Table 1.

**Table 1:** Summary of the validation dataset.

ESCC	83
Tumor size in mm, < 5/5 ~ 10/10 ~ 20/20 <	3/20/32/28
Macroscopic type, 0-I, IIa/0-IIb, IIc	7/7
Depth of tumor^a^, EP-LPM/MM/SM1/SM2–3	52/12/2/7
Non-cancerous lesions	64
lesion size in mm, < 5/5 ~ 10/10 ~ 20/20<	14/31/16/3
LGIN/Atypical/Esophagitis/Papilloma/Others	9/10/24/1/20

*ESCC* esophageal squamous cell carcinoma, *EP* epithelium, *LPM* lamina propria, *MM* muscularis mucosa, *SM* submucosa, *LGIN* low-grade intraepithelial neoplasia.

^a^Ten ESCCs diagnosed by biopsy were excluded from analysis of invasion depth.

### Performance of the AI system versus the endoscopists

The AI system diagnosed 71 of 83 SCCs (85.5%) as cancers and 48 of 64 noncancers (75.0%) as noncancers. The diagnostic performance of the AI system and the endoscopists are shown in Table 2. Accuracy, sensitivity and specificity of the AI system were superior or comparable to that of the endoscopists, even that of the experts.

**Table 2:** Diagnostic performance of the AI system and the endoscopists.

	AI system [95% CI]	All endoscopists (n = 19) (average) [95% CI]	Experts (n = 13) (average) [95% CI]	Non-experts (n = 6) (average) [95% CI]
Accuracy	80.9% (119/147) [73.6–87.0]	69.2% [66.6–71.9]	69.9% [66.5–73.2]	67.9% [61.9–73.9]
Sensitivity	85.5% (71/83) [76.1–92.3]	67.5% [61.9–73.1]	68.5% [60.9–76.1]	65.5% [55.0–75.9]
Specificity	75.0% (48/64) [62.6–85.0]	71.5% [62.6–80.3]	71.6% [59.7–84.8]	71.1% [53.5–88.7]
Positive predictive value	81.6% (71/87) [71.9–89.1]	76.1% [72.8–82.5]	78.4% [72.0–84.8]	76.1% [66.1–86.0]

*AI* artificial intelligence, *CI* confidence interval.

### Subgroup analyses by pathological diagnosis and lesion size

Table 3 shows the accuracy of the AI system and the endoscopists with respect to pathological diagnosis. The AI system showed better accuracy than the endoscopists in all categories, especially in ESCCs. Moreover, the AI system correctly classified all ESCCs that invaded the muscularis mucosa or submucosa, whereas even the experts diagnosed some of these cancers as noncancerous lesions.

**Table 3:** Accuracy of the AI system and endoscopist by pathological diagnosis and size of lesion.

	AI system (n), [95% CI]	Endoscopists^a^		
		All [95% CI]	Experts [95% CI]	Non-experts [95% CI]
**By pathological diagnosis**				
ESCC: 83 cases	85.5% (71/83) [76.1–92.3]	67.5% [61.9–73.2]	68.5% [60.8–76.1]	65.5% [55.0–75.9]
pEP/LPM: 52 cases	77.4% (41/52) [65.3–88.9]	57.2% [50.6–65.6]	57.9% [48.8–69.5]	55.7% [42.6–68.9]
pMM/SM1/SM2: 21 cases	100% (21/21) [83.9–100]	89.0% [84.6–93.4]	89.7% [84.1–95.4]	87.3% [77.5–97.1]
Non-ESCC: 64 cases	75.0% (48/64) [62.6–85.0]	71.5% [62.6–80.3]	71.6% [59.7–83.6]	71.1% [53.5–88.7]
LGIN/atypical: 19 cases	68.4% (13/19) [43.5–87.4]	64.8% [55.9–74.3]	64.0% [51.4–77.3]	66.7% [52.0–81.3]
Esophagitis/papilloma/others: 45 cases	77.8% (35/45) [62.9–88.9]	74.3% [66.8–84.8]	74.9% [64.6–88.6]	73.0% [55.4–92.8]
**By size of the lesions**				
< 10 mm: 68 cases	72.1% (49/68) [59.9–82.3]	68.0% [64.2–73.4]	67.6% [62.2–75.3]	68.9% [61.4–76.3]
≥ 10 mm: 79 cases	88.6% (70/79) [79.5–94.7]	70.3% [67.2–74.5]	71.8% [68.2–77.1]	67.1% [59.8–74.4]
≥ 20 mm: 31 cases	96.8% (30/31) [83.3–99.9]	82.7% [78.3–87.4]	84.1% [78.5–90.2]	79.6% [70.1–89.0]

*AI* artificial intelligence, *CI* confidence interval, *ESCC* esophageal squamous cell carcinoma, *EP* epithelium, *LPM* lamina propria, *MM* muscularis mucosa, *SM* submucosa, *LGIN* low-grade intraepithelial neoplasia.

^a^Percentages shown are averages.

Similarly, Table 3 shows the accuracy with respect to lesion size. The AI system also showed better accuracy than the endoscopists in these categories. In addition, the AI system correctly classified 96.8% of lesions ≥ 20 mm.

### Characteristics of the lesion for which the AI system had better accuracy than the endoscopist

Table 4 shows the characteristics of nine lesions that the AI system classified correctly but less than 30% of the endoscopists could. Eight of them were cancerous, and six of nine lesions were 10 mm or more in size. It revealed that the AI system could correctly classify ESCCs that the many endoscopists judged as non-ESCCs, and it might have more advantage for larger lesions.

**Table 4:** Lesions correctly classified by the AI system but by less than 30% of the endoscopists.

	ESCC	Non-ESCC	Total
< 10 mm	3	0	3 (33.3%)
10 mm ≤	5	1	6 (66.7%)
Total	8 (88.9%)	1 (11.1%)	9

*ESCC* esophageal squamous cell carcinoma.

**Figure 2:**
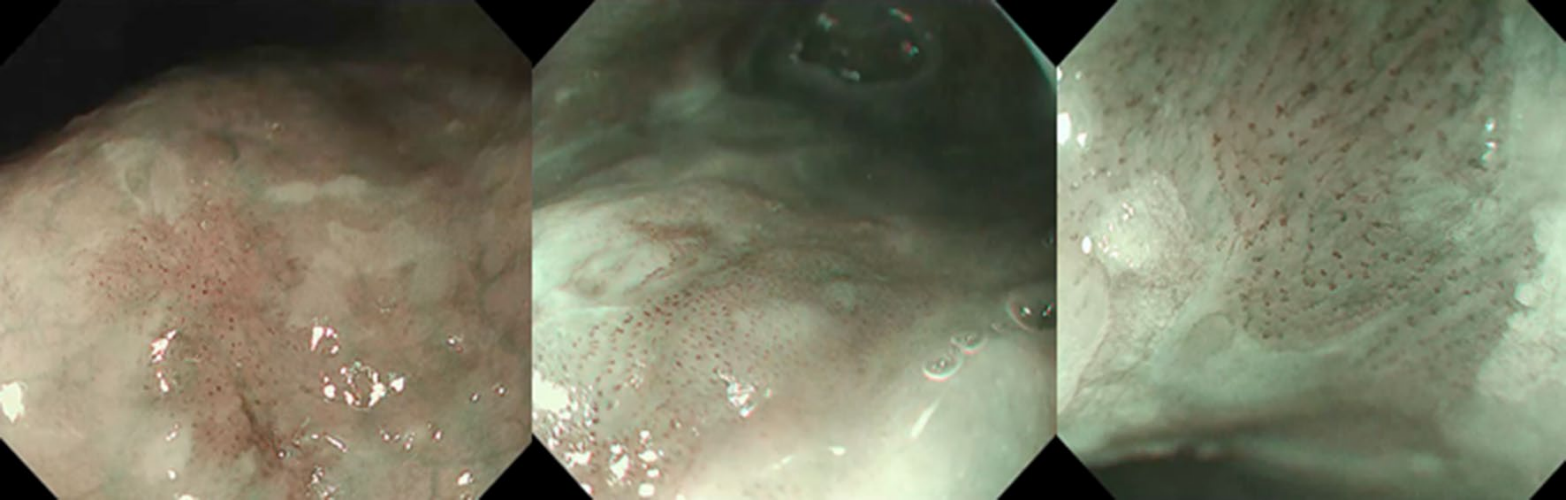
ESCCs cases correctly classified by the AI system but diagnosed as non-ESCCs by more than 70% of the endoscopists. These lesions showed fainter background coloration and slightly dilatated intrapapillary capillary loops.

For example, Fig. 2 shows the ESCC cases that the AI system classified correctly although more than 70% of the endoscopists diagnosed them as non-ESCC. These lesions showed faint background coloration and slightly dilatated intrapapillary capillary loops. They had been treated by ESD, and histologically diagnosed as ESCC.

## Discussion

In this study, we confirmed the high accuracy of our AI system, which was superior to the endoscopists, including the experts. Among various diagnostic parameters, our AI system showed a sensitivity that was about 20% higher than the experts. In addition, our AI correctly diagnosed eight cancers that were not correctly diagnosed by the majority (> 70%) of endoscopists. Because of its high sensitivity, our AI system could reduce the number of cancers being overlooked.

In subgroup analysis divided by lesions’ size and cancer invasion depth, our AI showed better performance in all subgroups. In particular, the performance was better in clinically significant lesions such as pMM/SM1/SM2 cancers and lesions ≥ 10 mm. Although final diagnosis is made by confirming pathological results of biopsy specimens, accurate endoscopic diagnosis is important to avoid overlooking cancers and to make appropriate clinical decisions.

Classification of lesions by the AI system can be conducted using video or still images. Most AI systems use still images for classification^15,18,24^ and we are also planning to introduce a similar system to the practice. Therefore, in this study, we used still images for the evaluation of our AI system. Conversely, endoscopists can classify lesions based on magnified and non-magnified observations. We therefore used video images of non-magnified and magnified observation for the evaluation of endoscopists. We consider that our study simulates the use of the AI system in clinical practice and reflects its performance in comparison with endoscopists.

From these results, our AI system would be a useful tool for supporting diagnosis, as it has a higher sensitivity and comparable specificity for cancer than general endoscopists. In addition, considering that the accuracy of endoscopic diagnosis had been reported to be comparable with that of biopsy diagnosis^25^, our AI system might be implemented as an optical biopsy with a high quality of diagnosis. As shown in Table 3, our AI system had higher diagnostic accuracy for ESCCs (85.5%) than for non-ESCCs (75.0%). We think this might be because 84% of the lesions in the training data used for our AI system were ESCCs. We need to improve the accuracy for non-ESCCs by using a training dataset containing more non-ESCC lesions.

We used the BiT learning system for our AI system. This is quite simple and has scaled-up pre-training. All BiT models consist of a customized vanilla ResNet-v2 architecture. In the architecture, all Batch Normalization layers are replaced with Group Normalization, and Weight Standardization is inserted into all convolutional layers.

This study has several limitations. First, we excluded four cases from the validation dataset where the pathological diagnosis was inconclusive because we could not determine whether such lesions should be regarded as cancer in the analysis. Second, esophago-gastric junctional cancers (E/J cancer), including Barrett's adenocarcinoma, were not included in this study. Because endoscopic findings of E/J cancer are quite different from ESCC, creation of a specialized AI system for E/J cancer is necessary. In this study we focused on the evaluation of ESCC.

In conclusion, our AI system showed higher accuracy than endoscopists for classifying ESCC and noncancerous lesions by ME in a situation simulating clinical use of the system. This system may therefore provide valuable support for endoscopists.
